# Macrophage-tregs crosstalk: the “hub” of the immune network in MASLD

**DOI:** 10.3389/fimmu.2025.1680878

**Published:** 2025-11-27

**Authors:** Huihui Zhao, Weili Wang, Pengchao Zhu, Zhaohong Shi

**Affiliations:** 1 College of Traditional Chinese Medicine, Hubei University of Chinese Medicine, Wuhan, China; 2 First Clinical Medical College, Anhui University of Chinese Medicine, Hefei, China; 3 Rehabilitation Department, Hubei Provincial Hospital of Integrated Chinese and Western Medicine, Wuhan, China; 4 Digestive System Department, Wuhan Hospital of Traditional Chinese and Western Medicine, Wuhan, China

**Keywords:** macrophage, regulatory T cells, MASLD, crosstalk, Kupffer cells

## Abstract

Metabolic dysfunction-associated steatotic liver disease (MASLD) is a globally prevalent metabolic disorder with a high average worldwide prevalence. It occurs more frequently in men than in women, and its incidence increases with age. MASLD can progressively advance to liver fibrosis, cirrhosis, and even hepatocellular carcinoma, while also elevating the risk of cardiovascular, renal, and other systemic diseases. Its pathological progression is closely associated with dysregulation of the hepatic immune microenvironment, in which aberrant crosstalk between Macrophages (Mø) and regulatory T cells (Tregs) serves as a central driving mechanism. Under physiological conditions, liver-resident Macrophages (Kupffer cells, KCs) and Tregs maintain immune homeostasis through a “complementary origin–spatial co-localization-molecular crosstalk” mechanism. In MASLD, KCs numbers decline while monocyte-derived Macrophages (MDMs) are abnormally recruited, giving rise to Macrophages with distinct phenotypes. Tregs influence the classical phenotypic differentiation of Macrophages. However, dynamic alterations in Treg abundance exhibit a “double-edged sword” effect. The disrupted crosstalk between KCs and Tregs involves dysregulated chemokine networks [e.g., c-x-c motif chemokine ligand 9 (CXCL9), c-c motif chemokine ligand 2 (CCL2)], cytokine interactions [e.g., interleukin-1β (IL-1β), transforming growth factor- Beta (TGF-β)], and signaling pathways such as beta-catenin (β-catenin) and notch homolog 1 (Notch1). Collectively, these alterations drive disease progression from steatosis to hepatitis and fibrosis. This review systematically summarizes the physiological mechanisms underlying Macrophages -Tregs crosstalk, its pathological dysregulation in MASLD, and the associated molecular networks, while proposing targeted therapeutic strategies based on disease stage.

## 1 Introduction

Metabolic dysfunction-associated steatotic liver disease (MASLD) is a form of metabolic liver disease characterized by hepatic steatosis that occurs in the absence of other specific liver disorders, though it may coexist with alcohol consumption. It has emerged as a major global health concern (1). In 2020, an international expert panel representing 22 countries officially renamed non-alcoholic fatty liver disease (NAFLD) as MASLD (2), emphasizing the central role of metabolic dysfunction in its pathogenesis (2). As global dietary patterns and socioeconomic conditions evolve, MASLD increasingly coexists with metabolic comorbidities such as obesity and diabetes, with its prevalence rising in parallel with these disorders (3). Epidemiological studies report a global average MASLD prevalence of approximately 30% (4), with regional variations: 17.31% in high-income regions (5), 32.45% in the United States (6), and 36.7% in China (7). The disease also exhibits demographic variation, with higher incidence in males than females and a notable age-related increase in prevalence (7, 8). Pathologically, MASLD progression involves hepatic metabolic derangements and chronic inflammatory responses. In severe cases, it may advance to metabolic dysfunction-associated steatohepatitis (MASH), liver fibrosis, and cirrhosis (9), potentially progressing to end-stage liver diseases such as MASLD-related hepatocellular carcinoma (1). These complications impose substantial burdens on both patient health and healthcare systems (4, 10). Furthermore, MASLD significantly increases the risk of cardiovascular and renal diseases (11, 12).

Maintenance of hepatic immune homeostasis relies heavily on the precise crosstalk between Macrophages and regulatory T cells (Tregs), and the disruption of this balance represents a key mechanism driving MASLD progression (13). Chronic inflammation is a major driver of MASLD advancement, primarily mediated by Macrophages and Tregs (14). Experimental studies have shown that, under physiological conditions, liver-resident Macrophages recognize pathogens to initiate innate immune defense, rapidly phagocytose foreign substances, and secrete cytokines as early warning signals (15–18), and this phenomenon is observed in both humans and animals (19). In mice, Tregs, in turn, sense inflammatory cues within the hepatic microenvironment through surface receptors and secreted cytokines, thereby constraining the excessive activation of effector T cells (20–22). In this “sensing-constraint” model, the delicate quantitative balance between the two classical Macrophages phenotypes (M1/M2: pro-inflammatory and anti-inflammatory) forms the basis for immune stability (23). However, in MASLD, this equilibrium is disrupted, leading to “bidirectional dysregulation.” On one hand, studies in MASLD mouse models show that Kupffer cells (KCs) numbers decline while monocyte-derived Macrophages (MDMs) are increasingly recruited (24, 25), a phenomenon that may also occur in humans (24). As Macrophages undergo phenotypic switching in response to environmental signals, their cytokine secretion patterns influence Tregs differentiation (13). Conversely, the expanding Tregs population exhibits dual, context-dependent effects (26). For example, in high-fat diet (HFD)-fed mice, increased hepatic Tregs alter Macrophages phenotype and function through multiple pathways (27). This dynamic transition from “homeostatic regulation” to “pathological drive” represents a critical mechanistic question that warrants further investigation in MASLD research.

As a globally prevalent chronic liver disease, MASLD progression is tightly linked to aberrant Macrophages-Tregs crosstalk. Previous studies have primarily examined Macrophages and Tregs individually, while the integrated mechanisms underlying their dynamic interaction during disease progression remain insufficiently characterized. This review systematically elucidates the biological basis of Macrophages-Tregs communication, highlights its pathological alterations in MASLD, and analyzes the underlying regulatory networks. We further propose stage-specific therapeutic strategies to advance understanding of MASLD pathogenesis and provide conceptual frameworks for both basic and translational research.

## 2 Physiological basis for macrophages-tregs crosstalk in the hepatic microenvironment

Under physiological conditions, hepatic immune homeostasis is maintained through a three-dimensional framework of “source complementarity-spatial co-localization-molecular interaction” between Macrophages and Tregs. These three layers function in a logically sequential and mutually reinforcing manner, forming a highly efficient immunoregulatory system.

### 2.1 Complementarity of origin and differentiation: homeostatic maintenance of cellular reservoirs

Macrophages and Tregs display pronounced complementarity in their cellular origins, ensuring both stability and functional diversity of the hepatic immune cell reservoir. KCs, the specialized liver-resident Macrophages, are constitutively localized within hepatic sinusoids, where they adhere to the surface of liver sinusoidal endothelial cells (LSECs). KCs origin involves two major sources. First, KCs primarily derive from yolk sac-specific progenitor cells during embryonic development, which colonize liver tissue and maintain population stability throughout life via self-renewal (28, 29). This “yolk sac origin-embryonic colonization-self-renewal” mechanism is highly conserved across mammals, including humans (30). Functionally, KCs serve as immune sentinels: in mice, they phagocytose foreign particles (31), while in human primary hepatic cells, they secrete anti-inflammatory mediators such as interleukin-10 (IL-10), thereby establishing the liver’s immunotolerant microenvironment (32, 33). Second, animal studies have shown that differentiation of bone marrow-derived MDMs—a process tightly regulated by local microenvironmental factors such as Macrophage Colony-Stimulating Factor—is minimal under physiological conditions, forming a KCs reservoir. Differentiated MDMs exhibit strong expression of transforming growth factor beta (TGF-β) activation-related pathways, maintain a proliferation index below 0.3% per week, and directly limit excessive expansion (34).

The tissue-resident Tregs populations in the liver are predominantly localized within the hepatic parenchyma and interstitial compartments (35). Under physiological conditions, Tregs utilize cytotoxic t-lymphocyte-associated protein 4 (CTLA-4) to competitively bind CD80/CD86 on LSECs, thereby preventing effector T cells from engaging these co-stimulatory ligands. This suppresses excessive effector T-cells activation, maintains local immune tolerance, and preserves hepatic immune homeostasis (35). Another subset of Tregs originates in the thymus. Following maturation, these cells enter the periphery as naive CD4^+^ T cells, which can differentiate into induced Tregs (iTregs) under the influence of the hepatic microenvironment (20, 27). The defining CD4^+^CD25^+^ fork head box p3 (FoxP3^+^) signature characterizes Tregs. Specifically, iTregs arise from naive CD4^+^ T cells through TGF-β-dependent differentiation, a process notably mediated by LSECs in the liver. These iTregs stably express FoxP3 yet retain numerical and functional plasticity, allowing adaptation to dynamic inflammatory cues (36).

The dual-origin paradigms of Macrophages—derived primarily from embryonic progenitors with bone-marrow supplementation—and Tregs—arising from both thymic derivation and peripheral induction—together establish a functionally complementary immune cell reservoir. This foundation provides the basis for their subsequent spatial co-localization and molecular interactions.

### 2.2 Spatial co-localization of macrophages and tregs: the foundation for precision crosstalk in the immune microenvironment

Building upon the stable cell pools formed through complementary origins, Macrophages and Tregs achieve spatial co-localization via chemokine-mediated regulation, establishing the physical conditions necessary for direct molecular interaction and functional synergy.

As liver-resident Macrophages, KCs are primarily located within hepatic sinusoids (HS) and adhere to LSECs. Tregs, by contrast, are concentrated in periportal regions and adjacent sinusoidal areas, displaying marked spatial overlap with KCs distribution patterns (37). This proximity enables their functional cooperation. Within this compartmentalized architecture, the HS, as key conduits for metabolic and immune exchange, are particularly susceptible to exposure from gut-derived antigens and metabolic stimuli. Under physiological conditions, KCs rapidly sense circulating exogenous molecules, such as microbial antigens from the gut, and initiate innate immune defense through efficient antigen capture (37). Tregs situated near KCs respond to these antigenic cues by secreting IL-10, thereby modulating local immune activation. Upon antigen uptake, KCs further promote the activation and expansion of antigen-specific Tregs. These activated Tregs, in turn, suppress the overactivation of KCs and MDMs, preventing excessive production of pro-inflammatory cytokines. This sequential process—antigen capture by KCs, activation of Tregs, and reciprocal regulation of Macrophages—constitutes a central mechanism by which the liver filters gut-derived antigens and protects the parenchyma from Macrophages-mediated injury (38). The periportal region functions as a critical immune hub, facilitating the recruitment of diverse immune cells. Here, the co-localization of MDMs, Tregs, and KCs collaboratively filters gut-derived antigens, mitigating chronic inflammation driven by the gut-liver axis (39). Even under pathological conditions, Tregs retain their protective function: by restraining immune intensity, they prevent secondary hepatic injury caused by excessive Macrophages activation (40). This compartmentalized collaboration, modulated by cytokines, chemokines, and metabolites, establishes a dynamic and context-dependent framework for immune cell interactions within the liver (20).

### 2.3 Molecular crosstalk between macrophages and tregs: from homeostatic collaboration to pathological disruption

Spatially co-localized Macrophages and Tregs execute the core functions of the “source complementarity–spatial co-localization–molecular interaction” framework through both direct intercellular binding and cytokine-mediated regulation, enabling precise and adaptable molecular crosstalk.

First, animal experiments have confirmed that direct molecular interactions potentiate immunosuppressive signaling. In pathological states, programmed death-1(PD-1) expressed on hepatic Tregs binds programmed death-ligand 1(PD-L1) on Macrophages, directly enhancing the immunosuppressive activity of Tregs. This interaction suppresses effector T-cell overactivation and helps preserve hepatic immune tolerance (41, 42). Additionally, antigen-specific interactions between Tregs and antigen-presenting cells (APCs)—where Treg T-cell receptors (TCRs) engage major histocompatibility complex class II(MHC-II) molecules on APCs—are essential for the targeted differentiation and activation of Tregs (43). Second, cytokine-mediated indirect regulation serves to balance the immune response. The co-secretion of IL-10 by Tregs and KCs generates a synergistic anti-inflammatory signal that protects hepatic tissue (44). Furthermore, the high-affinity interleukin-2 (IL-2) receptor α-chain (CD25) on Tregs competitively binds IL-2 in the hepatic microenvironment, depriving effector T cells of this critical proliferative signal and thereby inhibiting their expansion and secretion of pro-inflammatory cytokines such as tumor necrosis factor-alpha (TNF-α) and interferon-gamma (IFN-γ). Conversely, Macrophages secrete IL-2 and TGF-β to promote Tregs differentiation (41, 45). The maintenance of the anti-inflammatory phenotype of KCs is supported by the regulatory activity of other APCs. KCs sustain an M2-polarized state through toll-like receptor 4 (TLR4) signaling, which suppresses pro-inflammatory mitogen-activated protein kinase (MAPK) and nuclear factor kappa-light-chain-enhancer of activated B cells (NF-κB) pathways while upregulating anti-inflammatory mediators including TGF-β and IL-10 (41, 42, 45). Additionally, LSECs autonomously secrete TGF-β, thereby serving as key regulators of Tregs differentiation and function (36).

**Figure 1:**
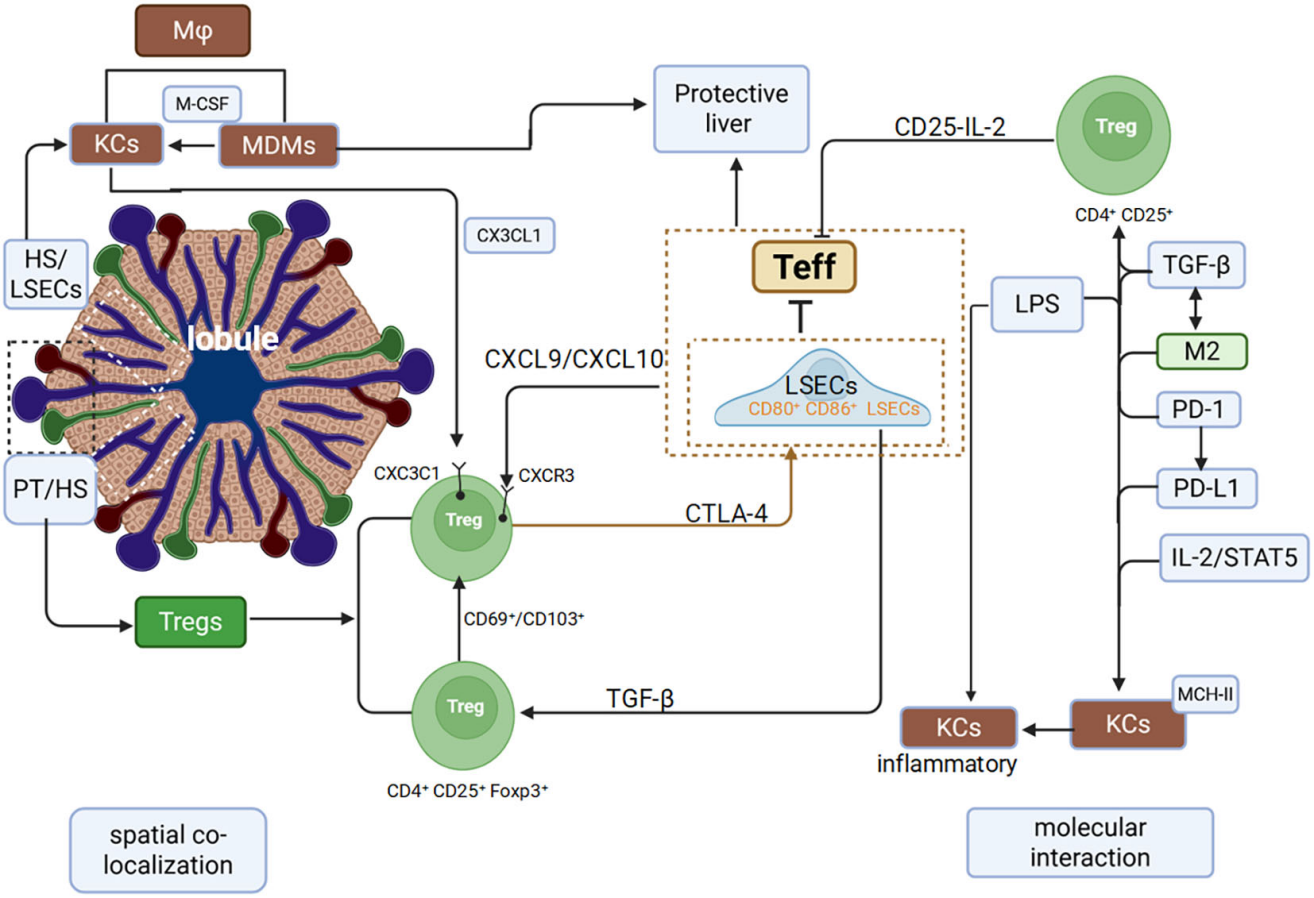
Spatial colocalization and molecular interactions between Macrophages and Tregs in the liver.

However, disturbances in metabolic, oxidative, and intestinal homeostasis can disrupt KCs-Tregs communication. Such dysregulation leads to pro-inflammatory MDMs infiltration and dysfunctional Tregs expansion, converting a homeostatic regulatory circuit into a pathological driver of inflammation (**Figure 1**).

## 3 Initiation of crosstalk imbalance in MASLD

### 3.1 KCs depletion and MDMs infiltration: early signals of crosstalk imbalance

In MASLD, the “source complementarity–spatial co-localization–molecular interaction” framework becomes progressively dysregulated. This cascade, from the destabilization of cellular reservoirs to disordered spatial localization and aberrant molecular communication, shifts Macrophages-Tregs crosstalk from homeostatic regulation to pathological activation.

**Figure 2:**
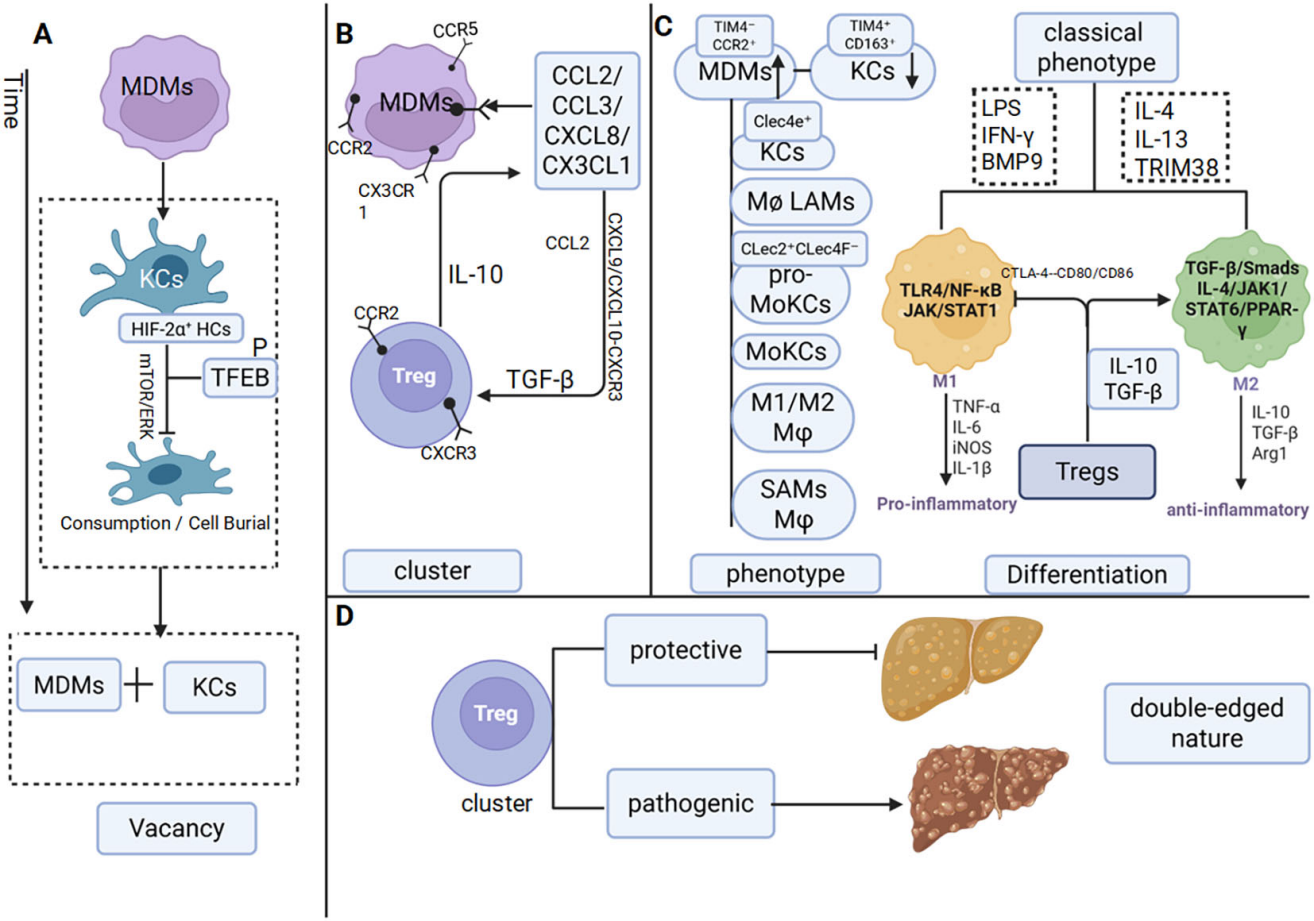
Macrophages and Tregs interact with each other in MASLD **(a)** Reduction in KCs. **(b)** Chemokine-mediated crosstalk between Macrophages and Tregs. **(c)** The phenotype and classical phenotypic differentiation of Macrophages are influenced by Tregs. **(d)** Dual roles of Tregs.

KCs depletion promotes MDMs recruitment and impairs KCs-Tregs anti-inflammatory coordination, thereby initiating the disruption of immunological crosstalk (46). The core mechanism of MDMs recruitment involves hepatic infiltration of circulating monocytes followed by their differentiation into phenotypically distinct Macrophages. In MASLD mouse models, depletion of c-type lectin domain family 4, member f (Clec4F^+^) T-cell Immunoglobulin and Mucin domain-containing protein 4 (Tim4^+^) KCs and expansion of MDMs subsets, including Clec4F^–^Tim4^–^ and Clec4F^+^Tim4^–^ populations resembling KCs, are observed. This phenomenon correlates with elevated Hypoxia-Inducible Factor-2 Alpha (HIF-2α) expression in KCs during Non-Alcoholic Steatohepatitis (NASH). HIF-2α activation of the mechanistic target of rapamycin (mTOR) and extracellular signal-regulated kinase (ERK) pathways induces TFEB phosphorylation, impairing KC phagocytic and efferocytotic functions and promoting apoptosis, as reflected by increased active caspase-3^+^ KCs (24, 25). Consequently, MDMs gradually become the predominant hepatic Macrophages population in MASLD (47). (**Figure 2a**).

Disruption of the embryonic origin stability of KCs and aberrant recruitment of MDMs directly undermine the cellular reservoir equilibrium central to the “source complementarity” framework.

### 3.2 Reciprocal regulation between macrophages and tregs recruitment

Following the imbalance in cellular origins, the nature of intercellular interactions also undergoes significant alteration. Cross-regulatory chemotactic signaling orchestrates the recruitment of MDMs and Tregs, synergistically reshaping the inflammatory microenvironment. A key mechanism driving the early progression from MASLD to metabolic-associated steatohepatitis (MASH) in mice involves the release of high mobility group box 1(HMGB1) from injured hepatocytes. By binding to TLR4 on KCs, HMGB1 activates the TLR4/ myeloid differentiation primary response 88 (MyD88)/NF-κB pathway, leading to KCs activation and the subsequent production of CCL2, with potential modulation of c-c motif chemokine ligand 3 (CCL3) and c-x-c motif chemokine ligand 8 (CXCL8). This chemokine milieu mediates the recruitment of Ly6C^+^ inflammatory monocytes and neutrophils, thereby initiating and sustaining NAFLD-associated inflammation (48–51). C-c motif chemokine ligand 2 (CCL2) directly promotes the recruitment of MDMs, including the triggering receptor expressed on myeloid cells 2 (Trem2^+^) subset, through interaction with c-c motif chemokine receptor 2 (CCR2) on Ly6C^+^ monocytes, while c-c motif chemokine ligand 5 (CCL5) acts synergistically with c-c motif chemokine receptor 5 (CCR5) in this process (52, 53). Additionally, IFN-γ-activated hepatic stellate cells (HSCs) express c-x3-c motif chemokine ligand 1 (CX3CL1), which undergoes proteolytic cleavage into a soluble form that binds to c-x3-c motif chemokine receptor 1 (CX3CR1) on monocytes, further enhancing MDMs recruitment (54). Conversely, genetic ablation of CCL2 or pharmacological blockade of CCR2/CCR5 provides reverse evidence supporting the critical role of this chemokine axis in Macrophages recruitment (52) (55).

CXCL9 and c-x-c motif chemokine ligand 10 (CXCL10) secreted by LSECs guide the migration of peripheral CD4^+^ T cells into the liver through CXCR3, where they differentiate into Tregs under TGF-β induction. Notably, monokine induced by gamma interferon (MIG)/CXCL9 downregulates CXCR3 expression on Tregs to fine-tune their immunosuppressive activity (56). Moreover, CCL2 derived from group type 1 innate lymphoid cell (ILC1s) not only recruits Tregs (40), but also establishes a feedback loop in which Treg-secreted IL-10 stimulates further chemokine production by immune cells (36), thereby establishing a regulatory circuit (**Figure 2b**).

### 3.3 Macrophages phenotypic differentiation and the regulatory role of tregs

#### 3.3.1 The phenotypic spectrum of macrophages in MASLD

In MASLD, Macrophages differentiation displays marked heterogeneity and dynamic plasticity. Firstly, it is the dual abnormality of the decrease in the number of KCs and their functional impairment. Under normal conditions, KCs express high levels of the markers CLEC4F, TIM4, and cluster of differentiation 163 (CD163). However, in MASLD mice, the number of TIM4^+^CD163^+^ KCs decreases significantly (57). Treatment with bisphosphonates depletes KCs, although resident KCs retaining phenotypic markers, along with CD68^+^ Macrophages, remain less affected. Concomitantly, mRNA levels of KCs-associated pro-inflammatory chemokines, including CCL2 and TNF-α, are significantly upregulated. These findings indicate that KCs numbers are reduced and that a subset of remaining KCs actively contributes to the pro-inflammatory process (58).

Secondly, there are changes in MDMs. They differentiate into pro-inflammatory MDMs in the early stage. When KCs depletion occurs, the number of TIM4^–^CCR2^+^ MDMs derived from Ly6C^+^ monocytes increases (57), with these MDMs distributed within the space of Disse and around the central vein. Ly6C^+^ Macrophages can further differentiate into short-lived Clec4e^+^ pro-inflammatory Macrophages (59). In MASLD mice, both lipid-associated Macrophages (LAMs) and monocyte-derived KCs (MoKCs) are markedly increased (60). In the early stages of MASLD, CLEC2^+^CLEC4F^–^ precursor MoKCs (pre-MoKCs) are recruited, expressing CX3CR1 and later differentiating into MoKCs that primarily localize within hepatic sinusoids (61). LAMs exhibit pro-inflammatory properties in the early stage and pro-fibrotic properties in the late stage. In mice fed HFD diet, hepatic F4/80^hi^CD11b^1n^TIM4^−^ CX3CR1-high LAMs are early-recruited subsets that express high levels of CX3CR1, CCR2, and Trem2 but low levels of CD63 and glycoprotein non-metastatic melanoma protein b (Gpnmb). Conversely, LAMs with low CX3CR1/CCR2 expression and high Trem2, CD63, Gpnmb, and secreted phosphoprotein 1 (Spp1) expression preferentially accumulate in HSC-activated regions, promoting the progression of liver fibrosis (47). Analysis of human clinical samples further reveals that the proportion of M1 Macrophages (CD86^+^, TNF-α^+^) is increased, whereas M2 Macrophages (CD206^+^) are reduced in MASLD patients (62).

Therefore, the differentiation of macrophages in MASLD is a complex process involving ldquo;KCs functional dissociation - MDMs subset specialization - dynamic functional transition (59, 63), and their phenotypes and functions exhibit high heterogeneity and plasticity.

#### 3.3.2 Classical phenotypic differentiation of macrophages and its regulation by tregs

M1 Macrophages are closely associated with the initiation of inflammation (64). Under stimulation by lipopolysaccharide (LPS) and IFN-γ, Macrophages polarize toward the M1 phenotype, secreting pro-inflammatory mediators such as Interleukin-1 Beta (IL-1β), TNF-α, and inducible nitric oxide synthase (iNOS), while activating the TLR4/NF-κB and janus kinase (JAK) /signal transducers and activators of transcription (STAT) signaling pathways (65). During early steatosis, lipid-laden hepatocytes release free fatty acids (FFAs) as damage-associated molecular patterns (DAMPs), which activate the NF-κB pathway through the TLR4/ myeloid differentiation 2 (MD-2) /Cluster of Differentiation 14 (CD14) complex on Macrophages, inducing pro-inflammatory cytokine secretion and promoting the differentiation of MDMs into M1 Macrophages (66–68). Studies using primary human hepatocytes show that BMP9 overexpression in NASH upregulates TLR4 expression on Macrophages surfaces (69). Sustained accumulation of M1 Macrophages in the human liver drives inflammation, disrupts lipid metabolism, and promotes hepatic fibrosis, thereby accelerating NASH progression (70). Tregs antagonize the pro-inflammatory activity of M1 Macrophages through multiple mechanisms. Animal studies have demonstrated that, in MASLD mice modeled with CCL2, hepatic Tregs directly inhibit M1 Macrophages activation via IL-10 secretion (40). Conversely, depletion of Tregs results in a significant increase in hepatic M1 Macrophages, further supporting their suppressive role (71).

In the MASLD liver, Tregs also promote Macrophages polarization toward the M2 phenotype. Upon liver injury, TGF-β secreted by Tregs suppresses Macrophages-derived pro-inflammatory cytokines, including TNF-α and IL-1β (72), while IL-10 from Tregs directly facilitates the M1-to-M2 phenotypic switch (40). M2 Macrophages polarization is further induced by IL-4 and IL-13 (73) through activation of the JAK/STAT6 signaling pathway (65, 74). The IL-4/JAK1/STAT6/peroxisome proliferator-activated receptor gamma (PPAR-γ) axis plays a key role in enhancing IL-4-driven M2 polarization (74). Notably, treatment with a PPAR-γ agonist increases splenic Tregs populations and elevates hepatic IL-10 levels in HFD-fed mice (75) (**Figure 2c**).

When the number of Tregs increases, the IL-10 they secrete inhibits the polarization of M1 Macrophages. Additionally, IL-10 and TGF-β synergistically promote the polarization of M2 Macrophages. This regulatory mechanism plays a crucial role in regulating the progression of inflammation and fibrosis during the pathological process of MASLD.

### 3.4 Dynamic imbalance of tregs quantity: a double-edged sword

In MASLD patients, the total number of intrahepatic CD4^+^ T cells decreases, whereas Tregs undergo significant expansion. This trend is corroborated in animal models, where IL-10 expression by Tregs is also markedly elevated (76). The expansion of intrahepatic Tregs does not arise solely from local proliferation but rather reflects enhanced recruitment and accumulation of peripheral Tregs due to alterations in the hepatic microenvironment. For example, animal studies have shown that amphiregulin (Areg)-producing Tregs are enriched in the livers of both mice and humans with NASH, where they contribute to tissue repair. Paradoxically, deletion of Areg in myeloid cells attenuates liver fibrosis (77). These findings demonstrate that FoxP3^+^ Tregs in MASLD exert a pronounced “double-edged sword” effect during disease progression (26). In early-stage MASLD, intrahepatic Tregs expand through recruitment and activation (78), supporting immune equilibrium and metabolic homeostasis. However, as the disease progresses to MASH and fibrosis, inflammatory and metabolic alterations in the hepatic microenvironment impair the immunosuppressive capacity of Tregs (79). At this stage, the increased Tregs population paradoxically exacerbates disease progression. Adoptive T-cell transfer experiments directly demonstrate that Tregs aggravate hepatic steatosis, enhance lipid accumulation, and worsen metabolic dysregulation (80)(**Figure 2d**).

The increased number of Tregs is accompanied by functional heterogeneity, and they regulate inflammation and fibrosis through the secretion of cytokines and other substances, thereby exhibiting a double-edged sword property.

## 4 Impact of dysregulated macrophages-tregs crosstalk on MASLD progression

### 4.1 The vicious cyclical role of chemokines in MASLD

Data from human genetic databases on MASLD indicate that CXCL9, a pivotal chemokine, exhibits a strong positive correlation with M1 Macrophages activity in the liver (81). CXCL9 is significantly upregulated in hepatocytes of patients with MASH, and a comparable increase is observed in mice fed an methionine-choline-deficient diet (MCD) diet (82). Silencing the MIG/CXCL9 gene in MASH mice ameliorates disease pathology, likely by altering the Treg/ interleukin-17 (Th17) cell ratio (56), thereby establishing a vicious cycle in which elevated CXCL9 suppresses Tregs function and promotes Th17 expansion, ultimately amplifying inflammation. In ApoA4^−/−^ models, the proportion of CXCL9-high inflammatory Macrophages subsets (2-Macrophages-CXCL9) markedly increases, exacerbating intrahepatic inflammation (83), and supporting its potential as a biomarker of disease progression (84). In contrast to the pro-inflammatory role of CXCL9, CXCL4 (PF4) is highly expressed in Ncoa5^−^ Macrophages, where it not only promotes lipid accumulation by activating the PPAR-γ2 pathway in hepatocytes but also induces M2 Macrophages polarization and recruits Tregs (85). This dual activity contributes to an aberrant hepatic microenvironment characterized by “lipid accumulation promotion plus pathological immune suppression.” In CD4^+^ T cell-specific KLF10 knockout mice, Tregs from HFD-fed animals exhibit impaired migration toward CCL19 (due to reduced CCR7 expression) and decreased TGF-β3 secretion. This dysfunction leads to the pathological accumulation of Ly6C^+^ high pro-inflammatory Macrophages and the formation of crown-like structures (CLSs) in adipose tissue (86).

**Figure 3:**
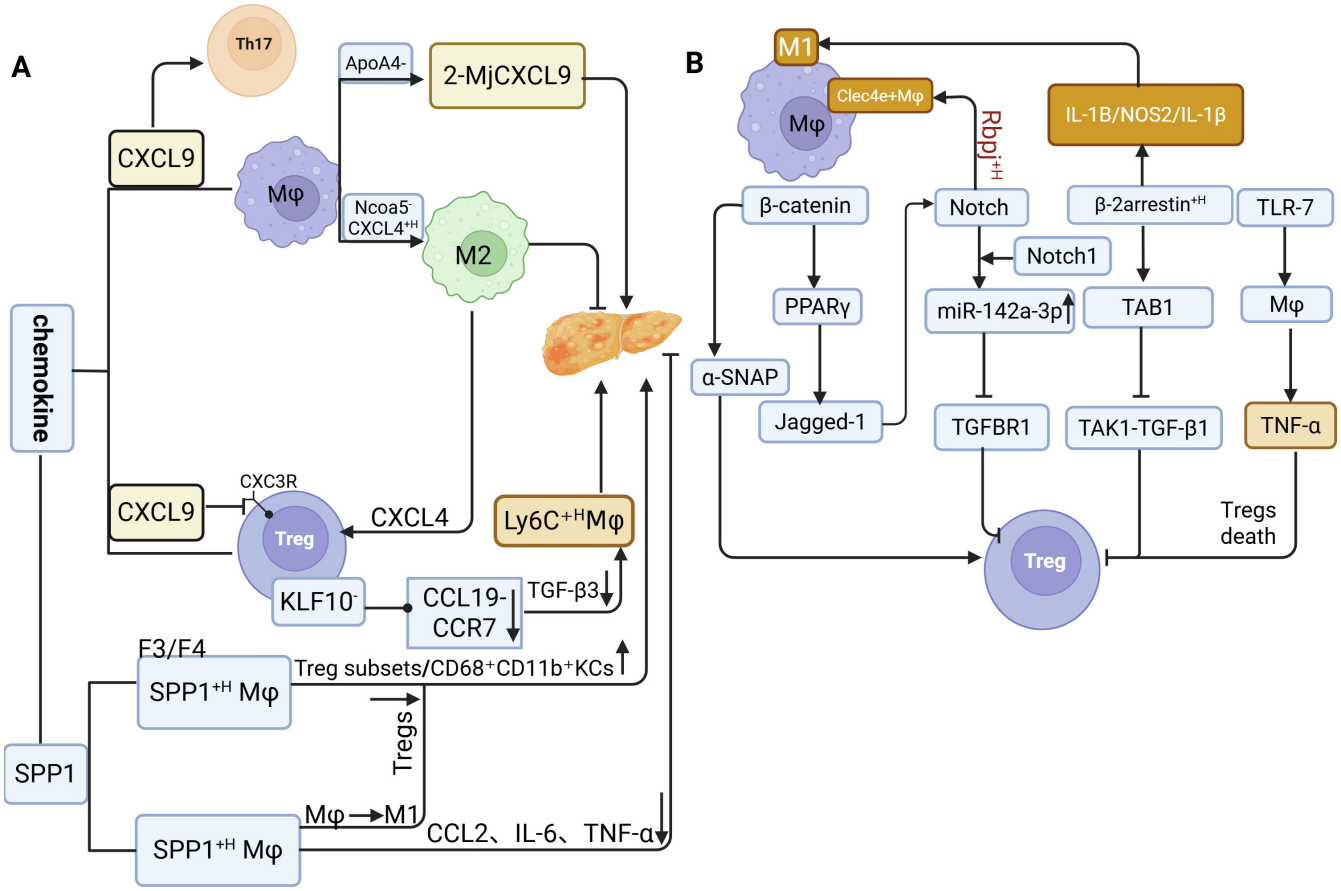
The molecular mechanisms underlying the interaction between Macrophages and Tregs. **(a)** Role of chemokines in regulating Macrophage-Treg crosstalk during MASLD progression. **(b)** Macrophages and Tregs promote MASLD through signaling pathways.

SPP1 exerts complex, bidirectional regulatory effects within the dynamic Macrophages-Tregs crosstalk network, displaying context-dependent roles that correlate with MASLD progression and immune infiltration (83, 84). In both NASH patients and murine models, SPP1^+^ -high Macrophages show an inverse correlation with pro-inflammatory genes such as CCL2, interleukin-6 (IL-6), and TNF-α, and are associated with lower steatosis scores, suggesting a potential anti-inflammatory and hepatoprotective role for these hepatic Macrophages populations (87). Conversely, in advanced fibrosis (F3-F4), increased SPP1 expression coincides with enhanced infiltration of Tregs and CD68^+^CD11b^+^ KCs, implicating SPP1 in fibrotic progression via immune cell recruitment (88). Furthermore, chronic intermittent hypoxia exacerbates hepatocellular injury and fibrogenesis through SPP1-mediated M1 Macrophages polarization (89). In obesity-driven chronic inflammation, SPP1 deficiency increases Tregs proportions, indicating that SPP1 overexpression normally suppresses Tregs accumulation (61) (**Figure 3a**).

The high expression of SPP1 regulates the crosstalk balance between Macrophages and regulatory Tregs. In the early stage, it exhibits anti-inflammatory properties by downregulating chemokine expression; in the late stage, however, it exerts pro-inflammatory and pro-fibrotic effects. These findings demonstrate the dual nature of SPP1.

### 4.2 Cytokine crosstalk in the inflammation-fibrosis transition

#### 4.2.1 Cytokine imbalance-mediated tregs-macrophages crosstalk in MASLD inflammatory progression

Cytokines act as the central mediators of Macrophages-Tregs crosstalk and serve as molecular switches driving the transition from inflammation to fibrosis in MASLD. Their dysregulated network profoundly alters Macrophages-Tregs interactions, directly influencing disease trajectory (90, 91). As previously discussed, Tregs regulate Macrophages phenotypic plasticity, and both cell types are dynamically shaped by the surrounding cytokine milieu. In murine MASLD models, T cell-specific nuclear receptor subfamily 4 group a member 1/2 (Nr4a1/2) double knockout leads to a significant expansion of tissue-resident Tregs (CD44^+^CD62L^–^CD69^+^), accompanied by reduced levels of pro-inflammatory cytokines such as IFN-γ and IL-17, and diminished activation of inflammatory Macrophages (92). This attenuation of systemic inflammation is largely mediated by IL-10 secreted from Tregs, which promotes M2 polarization of Macrophages (40). Moreover, Tregs expansion directly suppresses Macrophages infiltration, resulting in a marked reduction of hepatic inflammation (93). Conversely, genetic ablation of Tregs (Foxp3^DTR^ mice) induces substantial hepatic infiltration of neutrophils and Macrophages (93), coupled with decreased Arginase-1 expression in M2 Macrophages and elevated levels of M1-associated mediators such as IL-1β and iNOS (71). Collectively, these findings demonstrate that reciprocal phenotypic regulation between Tregs and Macrophages is a key determinant of inflammatory progression during MASLD.

#### 4.2.2 Th17/tregs imbalance drives the inflammation-fibrosis cascade

Within the dysregulated Macrophages-Tregs interaction network, the Th17/Tregs ratio and functional disequilibrium serve as pivotal determinants of inflammatory amplification and fibrotic progression in MASLD (94). Clinical evidence indicates that NASH patients with NAS scores > 4 display both increased intrahepatic Foxp3^+^ Tregs populations correlating with inflammatory severity and expanded CD68^+^ Macrophages regions (95). Elevated Th17 cell frequency is a hallmark of disease progression (96). IL-17, a key Th17 effector cytokine, exacerbates hepatocyte lipotoxicity via JNK pathway activation and counteracts the protective effects of interleukin-22 (IL-22), thereby impairing Tregs-mediated immunosuppression (97, 98). Additionally, IL-17 disrupts insulin signaling to worsen steatosis and synergizes with FFAs to induce IL-6 production in both HepG2 cells and murine hepatocytes. The combination of IL-6 and TGF-β promotes Th17 expansion (99), establishing a self-perpetuating inflammatory loop. This cascade ultimately impairs the suppressive function of Tregs within the hepatic microenvironment (79).

#### 4.2.3 Dual role of tregs and TGF-β in MASLD: from immunosuppression to fibrosis

TGF-β, a pleiotropic cytokine, suppresses cytotoxic t lymphocyte (CTL), T Helper 1 Cell (Th1), and T Helper 2 Cell (Th2) differentiation while promoting peripheral Tregs generation, thus maintaining immune homeostasis (100). In the liver, TGF-β facilitates immunosuppression by enhancing Tregs recruitment via LSEC-derived signaling and promoting M2 Macrophages polarization through Tregs-derived IL-10, establishing TGF-β as a central mediator of Macrophages-Tregs crosstalk (40).

However, the activation of TGF-β is also a critical driver of hepatic fibrogenesis. It classically induces extracellular matrix (ECM) gene transcription in HSCs via the Smad2/3 pathway. Concurrently, its interaction with the unfolded protein response (UPR) further amplifies ECM synthesis and HSC activation, thereby accelerating fibrotic progression (101). Both *in vivo* and *in vitro* studies have shown that Tregs and M2 Macrophages synergistically promote excessive TGF-β expression (40, 102). leading to aggravated fibrosis. Selective depletion of Tregs reduces M2 Macrophages proportions in fibrotic livers and decreases TGF-β secretion, suggesting that Tregs enhance TGF-β production by driving KCs polarization toward the M2 phenotype, thus facilitating fibrosis (71). Moreover, integrin αvβ8, specifically expressed by Tregs, cleaves latent TGF-β; its upregulation in fatty liver-associated Tregs directly implicates them in exacerbating fibrosis via enhanced TGF-β activation (27).

**Figure 4:**
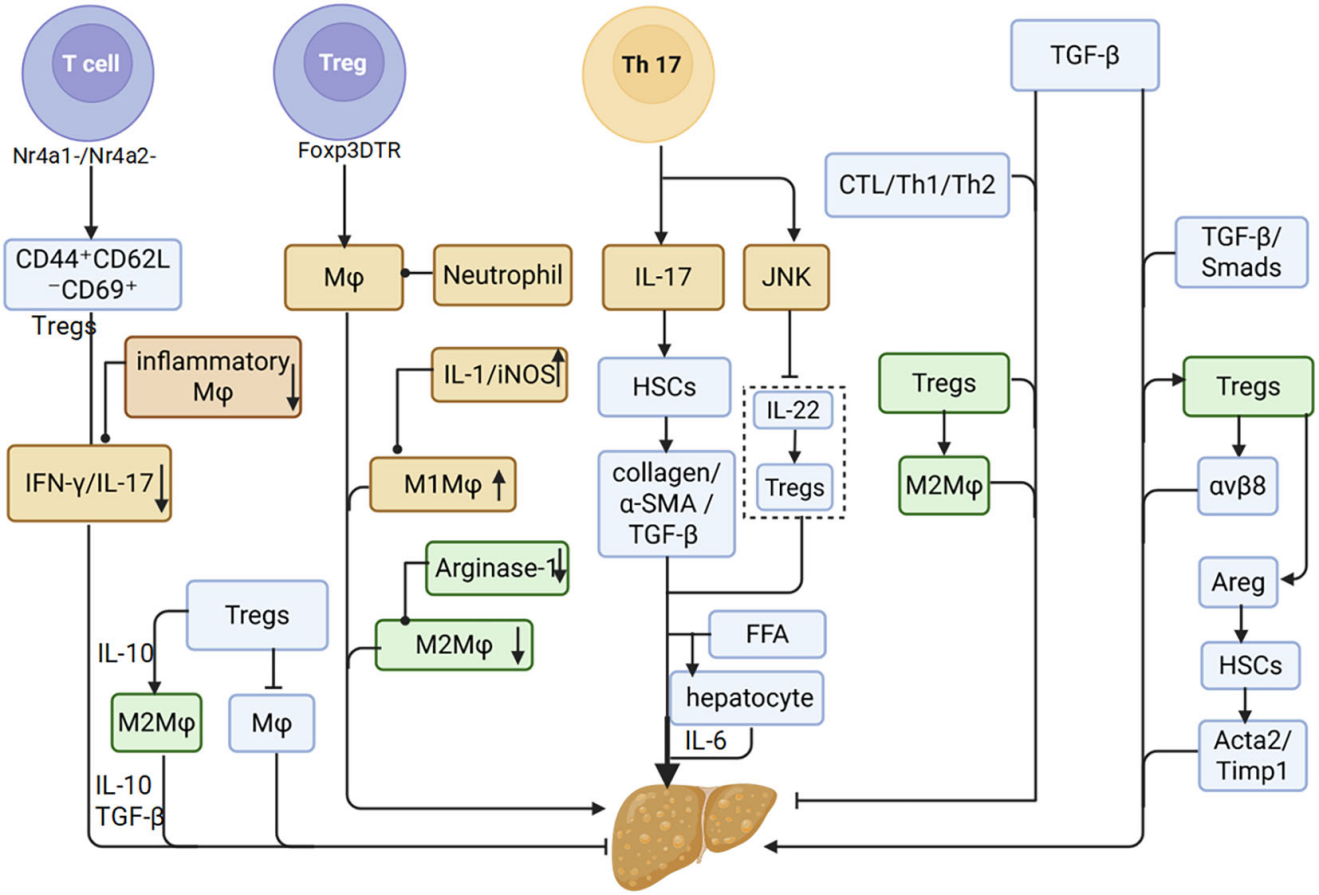
Macrophages and Tregs promote MASLD via cytokines and dual roles of Tregs and TGF-β in MASLD.

Tregs also promote fibrogenesis through Areg secretion in addition to TGF-β signaling (103). Tregs-derived Areg activates HSCs via epidermal growth factor receptor (EGFR) signaling, inducing myofibroblast transdifferentiation, enhancing collagen synthesis, and promoting ECM deposition. Conditional deletion of Areg in Tregs significantly reduces expression of HSC activation markers (α-smooth Muscle Actin 2 (α-SMA/Acta2) and Tissue Inhibitor of Metalloproteinases 1 (Timp1)) and attenuates fibrosis (77, 104) (**Figure 4**).

### 4.3 Crosstalk between signaling pathways

β-Catenin expression in MASLD displays stage-specific alterations distinct from those observed in healthy individuals. Studies demonstrate that β-catenin-related genes (TCF7L2, GLP1, AXIN2, FOSL1, WISP1) are suppressed in NASH patients (105), whereas β-catenin upregulation in infiltrating Macrophages promotes Tregs differentiation by regulating exosomal α-SNAP secretion (106). Furthermore, myeloid PTEN-mediated β-catenin activation induces FOXP3^+^ Tregs through the PPAR-γ/Jagged-1/Notch pathway while concurrently suppressing Th17 cell differentiation (107), a process likely coordinated through synergistic interaction with Notch1 (Notch1) signaling.

Notch1, the central transmembrane receptor of the Notch signaling pathway, also contributes to MASLD progression. In liver tissues from both MASLD patients and HFD-fed mice, Notch1 activation is markedly elevated, particularly in infiltrating Macrophages exhibiting β-catenin upregulation, and its activity shows a significant negative correlation with Tregs abundance. Macrophages-specific Notch1 knockout (Notch1^M-KO^) alleviates hepatic steatosis and normalizes Tregs frequencies through a mechanism dependent on Macrophages-derived exosomal miR-142a-3p, which targets TGF-β receptor 1 in T cells (13). In parallel, Rbpj, a key transcriptional regulator of Notch signaling, when deleted reduces the accumulation of pro-inflammatory Clec4e^+^ Macrophages and diminishes the production of inflammatory cytokines (59).

Beta-arrestin 2 (β-arrestin2) further modulates Macrophages polarization in the pathogenesis of MASH. In MASH patients, β-arrestin2 expression in hepatic CD68^+^ Macrophages and circulating monocytes positively correlates with hepatic steatosis severity and the expression of M1 markers (IL-1β, NOS2). Its knockdown suppresses M1 polarization, decreases pro-inflammatory IL-1β secretion, and increases anti-inflammatory IL-10 levels (108). Mechanistically, β-arrestin2 competitively binds tak1-binding protein 1 (TAB1), thereby inhibiting formation of the transforming growth factor-β-activated kinase 1 (TAK1)/ Transforming Growth Factor Beta 1 (TGF-β1) complex (109). Given that TGF-β1 is crucial for Tregs differentiation, this interference indirectly impairs Tregs development (101).

In addition, toll-like receptor 7 (TLR7) signaling regulates TNF-α secretion by KCs in MCD diet-induced MASLD mice, promoting Tregs apoptosis. Conversely, TLR7 knockout or TLR7 antagonist treatment restores Tregs proportions, mitigates intrahepatic inflammation, and reduces hepatic steatosis (110) (**Figure 3b**).

## 5 Stage-specific targeted therapeutic strategies for MASLD

Given the heterogeneity and dynamic progression of MASLD, therapeutic strategies targeting Macrophages-Tregs crosstalk must be tailored to specific disease stages. In the simple steatosis stage, dysregulated lipid metabolism and pro-inflammatory factor secretion by KCs dominate (111, 112). In contrast, during the MASH to fibrosis transition, infiltration of pro-inflammatory Macrophages phenotypes and an increase in Tregs abundance trigger activation of pro-inflammatory and pro-fibrogenic gene programs (113, 114). Consequently, the therapeutic focus differs across these stages.

Thus, pathology-stratified, stage-specific treatments represent a critical direction for future clinical translation. The following sections outline potential therapeutic strategies targeting Macrophages-Tregs crosstalk according to MASLD progression.

### 5.1 Synergistic regulation of macrophages and tregs to delay MASLD: for the simple steatosis stage

Early modulation of chemokine activity can ameliorate Macrophages-Tregs crosstalk, thereby delaying MASLD progression. The dual CCR2/5 inhibitor cenicriviroc reduces serum alanine aminotransferase (ALT) levels by shifting Macrophages polarization from Ly6C^hi^ to Ly6C^med^ subsets, concurrently suppressing pro-inflammatory cytokines (TNF-α, IL-1β, IL-6) and T-cell activation markers (IL-2, CD25^+^) (115).

Astaxanthin exerts multi-target protective effects in MASLD. It downregulates lipid metabolism genes (Sterol Regulatory Element-Binding Protein 1c (SREBP1c), FAS, CD36) to decrease hepatic triglyceride (TG), total cholesterol (TC), and non-esterified fatty acid (NEFA) accumulation. Simultaneously, it inhibits the JNK/p38 MAPK/NF-κB pathway, reducing F4/80^+^ Macrophages infiltration and pro-inflammatory cytokine expression (TNF, IL-6). Astaxanthin also promotes Macrophages polarization toward the M2 phenotype, upregulates CD11c^–^CD206^+^, Cd163, and IL-10, and decreases CD4^+^ T-cell infiltration by 54%. Clinical studies have confirmed that astaxanthin alleviates hepatic steatosis and improves NAS scores in NASH patients (116).

### 5.2 Modulation of β-catenin signaling in macrophages to delay MASLD progression: for the MASH stage

Therapeutically targeting the β-catenin signaling pathway may disrupt MASLD pathogenesis and represents a promising clinical strategy. The Wnt/β-catenin pathway plays a central regulatory role in MASLD. Studies show that CXXC5 expression is markedly elevated in both NASH patients and mouse models, while KY19334, a small-molecule activator, enhances Wnt/β-catenin signaling to suppress hepatic lipogenic genes (PPAR-γ, CEBPA), reduce hepatic infiltration of F4/80^+^ and Cd11b^+^ Macrophages, and downregulate inflammatory and fibrogenic markers (TNF-α, Mcp1, α-SMA, Col1a1), exhibiting superior efficacy to current treatments (105). Activation of the Wnt/β-catenin pathway also protects the gut vascular barrier against *Escherichia coli* NF73-1-induced disruption, preventing bacterial translocation and mitigating HFD-induced steatosis (117). Additionally, microRNA-21 antagonism enhances β-catenin signaling by upregulating Low-Density Lipoprotein Receptor-Related Protein 6 (LRP6), downregulating Glycogen Synthase Kinase-3 Beta (GSK3β), and increasing β-catenin stability, thereby improving lipid metabolism and reducing TG, TC, ALT, and aspartate aminotransferase (AST) through SREBP1c/ fatty acid synthase (FAS) inhibition and amp-activated protein kinase α subunit (AMPKα)/CPT1α activation (118). The glucagon-like peptide-1 receptor (GLP-1R) agonist Exendin-4 further modulates FABP1/FOXA1 expression via a Wnt/β-catenin-dependent mechanism, influencing lipid synthesis (SREBP-1/PPAR-γ) and very low-density lipoprotein (VLDL) secretion (ApoB) (119).

Treatment with PRI-724, a β-catenin/CBP inhibitor, significantly ameliorates hepatic steatosis and fibrosis in MASLD mouse models, evidenced by increased hepatic Marco^+^Mmp9^+^Cd68^+^ KCs and reduced levels of ALT, Mac-2 bp, and fibrotic markers (collagen I/III, α-SMA) (120). Similarly, the A3AR antagonist FM101 promotes lysosomal degradation of A3AR via β-arrestin2, effectively improving liver injury by reducing ALT/AST/cholesterol and downregulating fibrogenic (Col1a1, Col4a1, Lox, Timp1) and pro-inflammatory genes (TNF-α, IL-1β, CCL2/3), while inhibiting JNK/ERK/NF-κB and other key inflammatory pathways (121).

### 5.3 Targeting the tregs-Th17 balance for immunomodulation to delay MASLD: for the fibrotic phase

Regulating the Tregs-Th17 balance is a key immunomodulatory strategy to mitigate MASLD progression. Oral administration of the OKT3 antibody increases CD4^+^LAP^+^ and CD4^+^CD25^+^LAP^+^ Tregs populations, elevates serum TGF-β, significantly reduces AST, and improves insulin resistance and hepatic injury (122). Inhibition of IL-17 or IL-6 restores Th17/Tregs balance; IL-17 neutralization reduces ALT levels and hepatic inflammation via suppression of the JNK/NF-κB pathway (99). Polyenyl phosphatidylcholine (PPC) exerts therapeutic effects by downregulating Th17-associated factors ( retinoic acid-related orphan receptor γt (RORγt), STAT3, IL-6) and pro-inflammatory cytokines (TNF-α, TGF-β), while modulating the RORγt/FoxP3 ratio, leading to improvements in ALT/AST and lipid metabolism (TG/CHOL) (123). Ursolic acid alleviates MASLD in HFD-induced models by inhibiting Th17 differentiation through the SPP1-ITGB1/CD44-ERK pathway, thereby reducing TG, TC, ALT, AST, IL-17A, TNF-α, and IL-6, and diminishing hepatic lipid deposition. It also decreases fibrotic markers (α-SMA, collagen I, fibronectin) and extracellular matrix accumulation, alongside a pronounced reduction in IL-17A^+^CD3^+^CD4^+^ (Th17) cells in the liver (124).

Targeting Notch1 signaling can further coordinate Macrophages polarization and Tregs function. Notch1^M-KO^ alleviates steatosis and restores Tregs proportions (13). Quercetin suppresses Notch1 expression, reducing F4/80^+^ and CD68^+^ Macrophages infiltration, downregulating M1 markers (CD11c, IL-12, IRF5) and pro-inflammatory cytokines (TNF-α, IL-1β, IL-6, monocyte chemoattractant protein-1 (MCP-1), NOS2), while lowering M2-associated proteins (Ym-1, CD163, Arg I) and fibrotic indicators (Col3α1, Col4α1, CTGF, TIMP-1) (125). (**Table 1**).

**Table 1:** Drugs and their mechanisms for MASLD intervention.

Drugs	Model construction	Target cell	Target	Signaling pathway	Result	Reference
β-catenin pathway						
KY19334	HFD+CCl_4_	Macrophages	CXXC5-Dvl	Wnt/β-catenin	PPAR-γ↓, CEBPA↓, F4/80^+^Cd11b^+^Macrophages↓, TNF-α↓, cp1↓, α-SMA↓, Col1a1↓	(105)
Cenicriviroc	Lieber-DeCarli	F4/80loCD11bhi Macrophages, KCs, CD3^+^ T cell, CD25^+^ T cell	CCR2, CCR5	CCR2/CCR5	ALT↓, TG↓, Hydroxyproline↓, Coll mRNA↓, TNF-α↓, IL-1β↓, IL-6↓, CCL2↓, F4/80loCD11bhi Macrophages↓, Ly6Chi Macrophages↓, IL-2↓, CD25+T cell↓, CCL2 ↓, CCL5↓	(115)
Astaxanthin	HFD	Macrophages / M, M2, T cell			TG↓, TC↓, NEFA↓, SREBP1c↓, Lxra↓, CHREBP↓, FAS↓, SCD1↓, CD36↓, AST↓, ALT↓, TBARS↓, TNF mRNA↓, IL-6 mRNA↓, IL-1β mRNA↓, CD4^+^T cell↓, α-SMA↓, Tgfb1↓, Col1a1↓, PAI-1↓	(116)
Wnt3a	HFD, NF73-1	IMVECs	Wnt/β-catenin	Wnt/β-catenin	NF73-1↓, β-catenin↑, Toll4↓	(117)
antagomir-21	MCD	hepatocyte	miR-21, LRP6	Wnt/β-catenin	miR-21↓, LRP6↑, GSK3β↓, β-catenin↑, TG↓, TC↓, LDL↓, ALT↓, AST↓, SREBP1c↓, FAS↓, AMPKα↑, CPT1α↑, NAS↓	(118)
Exendin-4	OA	HepG2	GLP-1R, FOXA1, FABP1	Wnt/β-catenin	PLIN2↓, PLIN3↓, SREBP-1↓, PPARγ↓, CPT1A↓, ACC↓, DGAT1↓, SCD1↓, ACADL↑, FABP1↓, FOXA1↓, FFA↓, ApoB↓, VLDL↓	(119)
PRI-724	CDAHFD	HSC CD68^+^KCs	CBP/β-catenin	CBP/β-catenin	ALT↓, Mac-2↓, Coll I ↓, Coll III ↓, α-SMA↓, Mmp8 mRNA↑, Mmp9 mRNA↑, Marco^+^CD68^+^ Mmp9 ↑, PDK4↓	(120)
FM101	FFD	MDM KCs	A3AR	β-arrestin2	ALT↓, AST↓, TC↓, Col1a1↓, Col4a1↓, Lox↓, Timp1↓, TNF↓, IL-1β↓, CCL2↓, CCL3↓, TNF-α↓, caspase-1↓, GSK3β↓, caspase-8↓	(121)
Regulating the Tregs/Th17 balance						
OKT3	NASH	Tregs	CD3	Tregs/TGF-β	CD4^+^LAP^+^and CD4^+^CD25^+^LAP^+^Tregs↑, TGF-β↑, AST↓, ALT↓	(122)
PPC	HFD	Tregs Th17	IL-6, RORγt, STAT3, FoxP3	Th17/Treg	NAS↓, ALT↓, AST↓, TG↓, CHOL↓, Th17↓, Tregs↑, TNF-α↓, TGF-β↓, IL-6↓, IL-17↓, IL-23↓, RORγt↓, STAT3↓, IL-6↓	(123)
Ursolic acid	HFD	Th17	CD3^+^CD4^+^IL-17A^+^	SPP1-ITGB1/CD44-ERK	CD3^+^CD4^+^IL-17A^+^↓, ALT↓, AST↓, IL-17A↓, TNF-α, IL-6↓, α-SMA↓, Collagen I ↓, FN↓	(124)
Synergistic regulation						
Quercetin	CCl_4_	Macrophages HSCs	Notch1	Notch	Col III↓, Col IV↓, Col3α1↓, Col4α1↓, CTGF↓, TIMP-1↓, desmin↓, F4/80^+^ Macrophages↓, CD68^+^Macrophages↓, CD11c↓, IL-12↓, IRF5↓, TNF-α↓, IL-1β↓, IL-6↓, MCP-1↓, NOS2↓, Ym-1↓, CD163↓, Arg I↓, Notch1↓	(125)

## 6 Summary and future perspective

The dynamic balance between Macrophages and Tregs is essential for maintaining hepatic immune homeostasis, and their aberrant crosstalk constitutes a central mechanism driving MASLD progression. This interplay involves Macrophages recruitment, phenotypic differentiation, and quantitative changes in Tregs, orchestrated through networks of chemokines, cytokines, and signaling pathways. Dysregulation across these levels promotes the transition from hepatic inflammation to fibrosis.

The heterogeneity of both Macrophages and Tregs poses significant challenges for targeted modulation in MASLD. Macrophages phenotypic plasticity is dynamically shaped by the hepatic microenvironment, whereas Tregs exhibit stage-dependent dual roles, rendering single-target interventions insufficient. Moreover, the convergence of multiple intersecting pathways (such as β-catenin and Notch1) raises the risk of off-target effects. A critical barrier in MASLD clinical translation is the difficulty of precisely staging patients due to a lack of specific biomarkers, coupled with a dynamic immune microenvironment that renders therapeutic effects highly variable. The current lack of stage-stratified treatment protocols remains a major impediment to progress.

Future breakthroughs will require single-cell resolution analyses to delineate cellular heterogeneity and the development of stage-specific combination therapies targeting key pathways such as Notch1 and IL-17 through cell-specific delivery strategies.

## Glossary

MASLD: Metabolic dysfunction-associated steatotic liver disease
NAFLD: Non-alcoholic fatty liver disease
Tregs: Regulatory T cells
KCs: Kupffer cells
MDMs: Monocyte-derived Macrophages
HFD: High-fat diet
LSECs: Liver sinusoidal endothelial cells
IL-10: Interleukin-10
TGF-β: Transforming growth factor Beta
CTLA-4: Cytotoxic t-lymphocyte-associated protein 4
CD80: Cluster of differentiation 80
CD86: Cluster of differentiation 86
FoxP3: Fork head box p3
CD25: Cluster of differentiation 25
HS: Hepatic sinusoids
PD-1: Programmed death-1
PD-L1: Programmed death-ligand 1
APCs: Antigen-presenting cells
TCRs: Treg T-cell receptors
IL-2: Interleukin-2
TNF-α: Tumor necrosis factor-alpha
IFN-γ: Interferon-gamma
TLR4: Toll-like receptor 4
NF-κb: Nuclear factor kappa-light-chain-enhancer of activated B Cells
CLEC4F: C-type lectin domain family 4, member F
TIM4: T-cell immunoglobulin and mucin domain-containing protein 4
HIF-2α: Hypoxia-inducible factor-2 alpha
NASH: Non-alcoholic steatohepatitis
mTOR: Mechanistic target of rapamycin
ERK: Extracellular signal-regulated kinase
MASH: Metabolic-associated steatohepatitis
HMGB1: High mobility group box 1
MyD88: Myeloid differentiation primary response 88
CCL2: C-c motif chemokine ligand 2
CCL3: C-c motif chemokine ligand 3
CXCL8: C-x-c motif chemokine ligand 8
Trem2: Triggering receptor expressed on myeloid cells 2
CCR2: C-c motif chemokine receptor 2
CCL5: C-c motif chemokine ligand 5
HSCs: Hepatic stellate cells
CX3CL1: C-x3-c motif chemokine ligand 1
CX3CR1: C-x3-c motif chemokine receptor 1
CXCL9: C-x-c motif chemokine ligand 9
CXCL10: C-x-c motif chemokine ligand 10
MIG: Monokine induced by gamma interferon
ILC1s: Type 1 innate lymphoid cell
CD163: Cluster of differentiation 163
FPC: Fructose: palmitate and cholesterol
